# Training needs analysis of surgical teams in Somaliland

**DOI:** 10.1093/bjs/znaf216

**Published:** 2025-12-10

**Authors:** Gerard McKnight, Hassan Ali Daoud, Rocco Friebel, Rachel Hargest

**Affiliations:** School of Medicine, Cardiff University, Heath Park, Cardiff, UK; Humanitarian Surgery Initiative, Global Affairs Department, Royal College of Surgeons of England, London, UK; Academic Department of Military Surgery & Trauma, Royal Centre for Defence Medicine, Edgbaston, Birmingham, UK; Global Surgery Policy Unit, LSE Health, London School of Economics and Political Science, London, UK; Suuban Center for Health System Strengthening, Hargeisa, Somaliland; Global Surgery Policy Unit, LSE Health, London School of Economics and Political Science, London, UK; Department of Health Policy, London School of Economics, London, UK; School of Medicine, Cardiff University, Heath Park, Cardiff, UK; Humanitarian Surgery Initiative, Global Affairs Department, Royal College of Surgeons of England, London, UK; Global Surgery Policy Unit, LSE Health, London School of Economics and Political Science, London, UK

## Abstract

**Introduction:**

Prioritizing resources is essential for low-income countries aiming to improve surgical systems effectively. Few validated tools exist to facilitate this. The authors aimed to address this through the novel application of an existing training needs analysis (TNA) tool to a surgical context in a low-income country.

**Methods:**

A questionnaire was designed as a mixed-methods, online survey to capture quantitative and qualitative data based on the Hennessy–Hicks training needs analysis (HHTNA) Questionnaire. The survey was distributed by collaborating organizations in Somaliland.

**Results:**

Responses were received from 41 anaesthesia providers (APs) and 69 surgical providers (SPs), giving a response rate of approximately 59% of APs, 33% of surgeons, and 21% of obstetricians in Somaliland. The HHTNA of APs highlighted that emergency front of neck access (cricothyroidotomy) was a ‘high intervention priority’ procedure among APs. Regional anaesthesia, medical management of co-morbidities, and anaesthesia in geriatric populations were also considered performance outliers and should also be the focus of further intervention. Importantly, mixed interventions were desired, indicating that training alone would be insufficient, and that improvements to the work situation also need to be addressed.

**Conclusion:**

This study has demonstrated that conducting a pragmatic TNA of the surgical team in a low-resource setting, such as Somaliland, is both feasible and can generate useful data to guide training and professional development.

## Teaser

Prioritizing resources is essential for low-income countries aiming to improve surgical systems effectively, yet few validated tools exist to facilitate this. This mixed-methods online survey was designed using existing methodology to conduct a training needs analysis (TNA) of surgical teams in Somaliland. The findings will help prioritize the educational needs of the existing workforce and has also demonstrated that a pragmatic, cost-effective TNA is feasible in low-income countries.

## Introduction

In 2015, the United Nations’ sustainable development goals (SDGs) produced a set of focused healthcare targets to be achieved by 2030^1^. Those that relate directly to surgical care include targets 3.1 ‘reduce maternal mortality’, 3.2 ‘end all preventable deaths under 5 years’, 3.4 ‘reduce mortality from non-communicable disease’, and 3.6 ‘reduce road injuries and death’. Addressing these targets requires the focused allocation of limited resources to improve existing health systems. However, there are limited tools in current practice that allow for rigorous assessments of the training needs of the existing workforce to prioritize resource allocation in support of the SDGs. The authors aimed to address this through the novel application of an existing training needs analysis (TNA) tool to a surgical context in a low-income country.

Somaliland is a ‘de-facto’ low-income country in the Horn of Africa that emerged from the Somali civil war in 1991 and is yet to be formally recognized by the international community. Despite the lack of international recognition, it has undergone the process of building the systems of state in a peaceful environment^2^. It has a population of approximately 4 million people, of whom 40% live in the capital city Hargeisa and 55% are estimated to be nomads^2,3^. It is the fourth poorest nation in the world, and with constant drought and famine has significant challenges with delivering health services^2,3^. Health services have been targeted towards the original millennium development goals and focused on reducing poverty, addressing communicable diseases, and reducing child and maternal mortality^2,4^.

The Somaliland surgical system includes 16 hospitals and requires strengthening in every facet, including the training and continued development of the workforce^3^. One recent analysis by Dahir *et al.*, using the WHO’s Surgical Assessment Tool, identified that Somaliland did not meet the Lancet Commission on Global Surgery (LCoGS) minimum standard in any of the six indicators^3,5^. This study highlighted that the density of surgeons, anaesthetists and obstetricians (SAO density) was 0.8/100 000 and the procedure volume was 368.8 procedures/100 000, significantly below the LCoGS benchmarks of 20 SAO providers and 5000 procedures per 100 000 population^3,5^. The same study identified 15 surgeons, 3 anaesthetists and 14 obstetricians working across the country.

As discussed in the paper by Qin *et al.* in this issue, there have been numerous educational initiatives to improve surgical training and expertise in low- and middle-income countries (LMICs), which usually involve taking courses which were designed by surgeons in high-income countries (HICs) for practice in HICs and exporting them directly to surgeons in LMICs. Somaliland has been a target country for various charitable and philanthropic educational initiatives that have aimed to bring HIC standards of surgical practice to hospitals and clinics there. However, there have been few studies asking clinicians in LMICs what training is required and how relevant further training would be to their role. This study was designed to start by approaching surgical providers (SPs) and anaesthetic providers (APs) in Somaliland to determine their learning requirements, rather than exporting a pre-existing course, with the hope that appropriate educational solutions can be produced in response. Therefore, the primary aims of this study were to identify the training needs of APs and SPs in Somaliland and to identify how technology could improve training. The secondary aims were to validate a novel surgical TNA survey and to demonstrate the utility of a pragmatic TNA in a low-income country.

## Methods

A questionnaire was designed as a mixed-methods, online survey to capture quantitative and qualitative data based on the Hennessy–Hicks training needs analysis (HHTNA) questionnaire^6^. The Good Reporting of a Mixed Methods Study guideline has been followed for reporting of results—see *Table S1*^7^.

Collaborating with multiple organizations is essential in any attempt to strengthen a surgical system, and this is especially true when working across international boundaries. This study was conducted as a collaboration between two groups:

- The Global Surgery Policy Unit (GSPU), a collaboration between the Royal College of Surgeons of England (RCS Eng) and the London School of Economics and Political Science.
- Partnerships for Surgical Systems Strengthening Somaliland (PaSSS Somaliland)—a collaboration between InciSioN Somaliland, Somaliland Medical Association, Somaliland Nursing Anaesthesia Network (SNAN), Somaliland Nursing & Midwifery Association, Somaliland Medical Laboratory Association, Hargeisa Group Hospital, Burco Regional Hospital, Medicine Africa and RCS Eng.

Participant recruitment was led by the Somaliland Medical Association and the SNAN. Jotform (Jotform Inc. San Francisco, USA) was used to deliver the survey and collect data in conjunction with MedicineAfrica (Oxford, UK). Inclusion criteria were APs or SPs currently practicing in Somaliland. APs and SPs were defined as any healthcare professional (with dedicated undergraduate training) providing anaesthetic or surgical care respectively. A separate survey was designed for SPs and APs.

Data were downloaded from Jotform and analysed in both Microsoft Excel (Microsoft^®^ Excel, Microsoft^®^, Version 16.80) and SPSS (IBM^®^ SPSS^®^ Statistics, Version 29). Quantitative data were analysed using descriptive statistics and means were compared using a paired *t*-test with statistical significance set at *P* of 0.05. Only the quantitative data have been reported in this article.

Participants were identified by the relevant partner organizations in Somaliland and targeted through emails, social media, and in-person approaches at their place of work. Incentives were offered to the partner organizations to encourage participation as part of the wider PaSSS Somaliland project.

Ethical approval was granted by both the London School of Economics and Political Science, Department of Health Policy and by the Somaliland Ministry of Health Development. Project oversight was undertaken by the GSPU.

The surveys assessed the training need in critical skills within each profession. The skills included were influenced by a literature review using peer-reviewed data or reference standards where available. The surveys were each tailored to the local requirement in Somaliland in agreement with PaSSS Somaliland.

The surgical skills included were based on the ‘basket’ of surgical procedures proposed by Odland *et al.* as a more granular version of the ‘bellwether procedures’ proposed by the LCoGS^5,8^. The anaesthesia skills assessed were based on the Safer Anaesthesia for Education (e-SAFE) modules designed by the Royal College of Anaesthetists, the Association of Anaesthetists of Great Britain & Ireland, the World Federation of Societies of Anaesthesiologists, and e-Learning for Healthcare^9–12^. Additionally, all APs were asked if they have access to accurate and reliable oxygen saturation monitors, as this is an area that was locally identified as a concern and cost-effective solutions are currently available^13^. All respondents were asked if they used the WHO Surgical Safety Checklist, as adherence to this checklist has been demonstrated to reduce mortality and morbidity rates from surgery across the world^14^.

The survey was trialled in English among a cohort of Somaliland healthcare professionals from each of the professional organizations to ensure that it was straightforward to complete, the format was easy to follow, and that it was relatively short. Minor amendments were made at this stage to improve clarity and to ensure it was relevant to the needs of the collaborating organiations. This initial pilot ensured that the survey showed good face validity and was practical within the local context.

Although the HHTNA has been well validated in the UK primary care nursing population, using it in a very different context with significant structural changes required validation of the modified version. Therefore, an analysis of the validity of the survey was undertaken as a secondary outcome of the study. Validation of this modified version of the HHTNA may encourage other researchers to use this tool in future.

Following the original Hennessy–Hicks methodology, an analysis of reliability was undertaken using a principal component analysis (PCA) and Cronbach's alpha^15,16^. The PCA was performed with Varimax rotation on each of the scales used in the survey. Additionally, the Kaiser–Meyer–Olkin (KMO) measure was used to estimate sampling adequacy.

## Results

### Anaesthesia providers

Responses were received from 41 APs, giving a response rate of 59% of APs in Somaliland. One of the responses was from an anaesthetist, giving a response rate of 33% of Somaliland anaesthetists. Of the 41 responses, 41% (17) were female and 59% (24) were male—see *Table 1*. The cohort was young, the majority (80%, 33) were aged between 25 and 34 years, and there were no respondents over age 44. There was one (2%) doctor, and all other respondents were nurses (2, 5%) or APs (38, 93%).

**Table 1:** Demographics of anaesthesia provider respondents

Demographic	Number	Percentage
**Age**		
18–24	2	5
25–34	33	80
35–44	6	17
45–54	0	–
55–64	0	–
65 or above	0	–
**Gender**		
Male	24	59
Female	17	41
**Profession**		
Anaesthesia provider	38	93
Nurse	2	5
Doctor	1	2
**Level of training**		
Student	6	15
Qualified health worker	33	80
Specialist (with postgraduate training)	2	5
**Experience**		
Less than 3 years	23	56
3–5 years	9	22
6–10 years	7	17
11–15 years	2	5
16–20 years	0	–
More than 20 years	0	–
**Location of training**		
Somaliland	41	100
Other	0	–
**Region of work**		
Marodi Jeh	23	56
Awdal	8	20
Togdheer	5	12
Sanaag	2	5
Gabiley	2	5
Sahil	1	2
Sool	0	–
**Workplace setting**		
Urban	27	66
Rural	14	34
**Workplace finance**		
Public/government facility	25	61
Private facility	12	29
Non-governmental organization or charity	4	10
**WHO level**		
Primary care/community facility	10	24
Secondary care/WHO Level 1 hospital	17	41
Tertiary care/WHO Level 2 or 3 hospital	14	34
**National Health Professions Commission (NHPC) licence**		
No	26	63
Yes	13	32
Don't know	2	5

Reliable oxygen saturation monitoring was available intraoperatively to 80% (33) of respondents. However, 7% (3) reported ‘sometimes’ having access and the same number (7%, 3) answered ‘no’. The WHO checklist was ‘always’ used by 21 (51%) of respondents, ‘often’ by 11 (27%), and ‘rarely’ by 9 (22%).

**Fig. 1:**
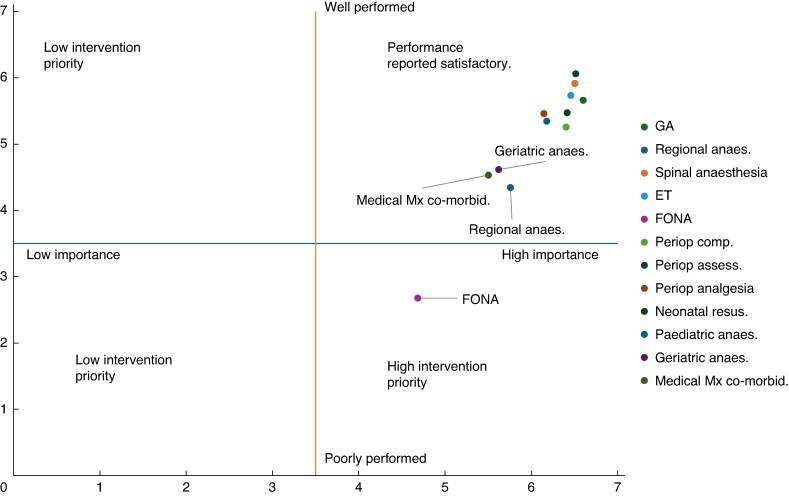
Quadrant graph displaying mean importance *versus* mean performance scores for each anaesthetic skill

The highest mean importance was reported in administration of general anaesthesia (6.60), followed by perioperative assessment of a patient undergoing surgery (6.51) and spinal anaesthesia (6.50). The lowest mean importance was found in emergency front of neck access (FONA or cricothyroidotomy) at 4.69, followed by medical management of medical co-morbidities (5.50) and anaesthesia in the geriatric population (5.62). The mean ratings for importance and performance of each skill are presented in *Table S2*. These same data are displayed in a quadrant graph format in *Fig. 1*, which demonstrates that emergency cricothyroidotomy (FONA) was reported as a high intervention priority according to the Hennessy–Hicks methodology. All other skills were reported as satisfactory.

The highest mean rating of importance for improving training alone was found in the management of perioperative complications (5.80) followed by paediatric anaesthesia (5.74) and FONA (5.63). The lowest mean rating was found in neonatal resuscitation (5.23), followed by the medical management of medical co-morbidities (5.29) and spinal anaesthesia (5.32). Further detail can be found in *Table S3*.

**Fig. 2:**
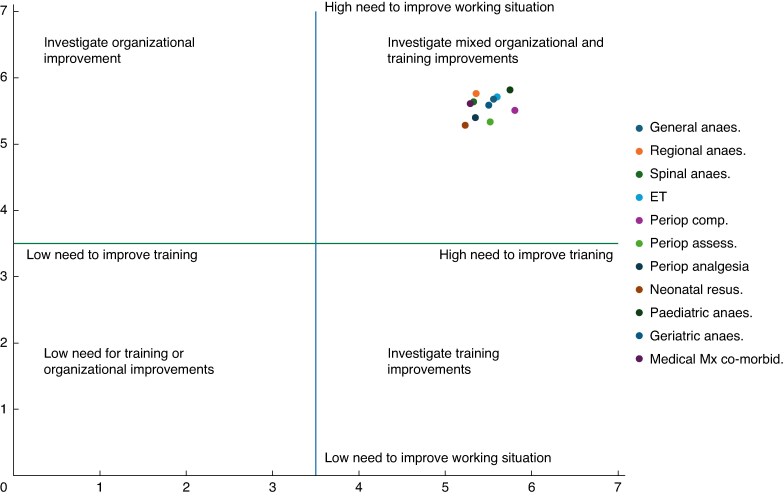
Quadrant graph displaying mean scores for need to improve training *versus* need to improve the work situation

The highest mean rating of importance for improving the work situation was found in paediatric anaesthesia (5.81), followed by regional anaesthesia (5.76) and endotracheal intubation (5.71). The lowest mean rating of importance for improving the work situation was found in neonatal resuscitation (5.28), followed by perioperative assessment (5.33) and FONA (5.38). These data are displayed in a quadrant graph in *Fig. 2*, which demonstrates that mixed organizational and training improvements were desired for all assessed skills.

Answers to the question ‘what technology is currently used?’ are displayed in Fig. S1. ‘Online lectures’ and ‘Watching video recording of an operation or procedure’ were the joint most popular responses, selected by 51% (21) of respondents. This was followed by ‘Sending clinical photographs or videos’ and various websites by 46% (19) of respondents. ‘Video calls to patients’ was the least frequently reported use of technology, with 19.52% (8) respondents.

### Surgical providers

Responses were received from 69 SPs, 57 (83%) of whom were doctors without specialist training, 5 (7%) were surgeons, and 5 (7%) were obstetricians. As there are no confirmed overall numbers of SPs in Somaliland, response rates have been subdivided into specialists, giving a response rate of 33% of surgeons and 21% of obstetricians. The demographics of respondents can be found in *Table 2*.

**Table 2:** Demographics of SP respondents in Somaliland (*N* = 69)

Demographic	Number	Percentage
**Age**		
18–24	4	6
25–34	59	86
35–44	6	9
45–54	0	–
55–64	0	–
65 or above	0	–
**Gender**		
Male	47	68
Female	22	32
**Profession**		
Doctor/physician	57	83
Surgeon (doctor with formal postgraduate training in surgery)	5	7
Obstetrician/gynaecologist	5	7
No answer	2	3
**Level of training**		
Student	2	72
Qualified healthcare professional	50	22
Specialist (with postgraduate training in your speciality)	15	3
No answer	2	3
**Experience**		
Less than 3 years	42	61
3–5 years	15	22
6–10 years	12	17
11–15 years	0	–
16–20 years	0	–
More than 20 years	0	–
**Location of training**		
Somaliland	60	87
Uganda	2	3
Egypt	1	1
Somalia	2	1
Ethiopia	2	3
No answer	2	3
**Region of work**		
Marodi Jeh	9	13
Awdal	44	64
Togdheer	7	10
Sanaag	2	3
Gabiley	1	1
Sahil	0	–
Sool	2	3
No answer	4	6
**Workplace setting**		
Urban	52	75
Rural	17	25
**Workplace finance**		
Public/government facility	41	59
Private facility	24	35
Non-governmental organization or charity	4	6
**WHO level**		
Primary care/community facility	23	33
Secondary care/WHO Level 1 hospital	33	48
Tertiary care/WHO Level 2 or 3 hospital	13	19
**NHPC licence**		
No	34	49
Yes	30	43
Don't know	3	4
No answer	2	3

The WHO Surgical Safety Checklist was used ‘always’ by 28% (19) of SP respondents, ‘often’ by 19% (13), ‘rarely’ by 26% (18), and ‘never’ by 17% (12).

The most frequently reported technology currently in use among SPs was ‘Video calls to colleagues’, ‘Sending clinical photographs or videos’ and ‘Online lectures’, each of which was reported by 26% (18) of respondents—see *Fig. S2*. Remote supervision of procedures was the least frequently reported use of technology, by 3% (2) of respondents.

Both ‘online lectures’ and ‘Webinars for training or continued professional development (CPD)’ were the most frequently response for the future use of technology, by 57% (39) of respondents. ‘Videoconferences’ were the second most frequently reported, by 46% (32). ‘Sending clinical photographs or videos’ was the least frequent response, by 23% (16).

## Reliability

A PCA was conducted and repeated for each of the scales with results in *supplementary material*, the Cronbach's alpha, standardized alpha and KMO measure are also displayed in *Table S4*. The KMO measure verified the sampling adequacy for the analysis of 0.694, which is above the acceptable limit 0.5, according to Kaiser and Rice^17^. Minimal differences were seen between the results for Cronbach's alpha and the standardized alpha, indicating comparable variance. These results suggest that the scales and results provided can be considered reliable.

### Overall SAO density

The responses have been used to estimate the SAO density in Somaliland at 1.77/100 000, which is far below the 20/100 000 target set by the LCoGS. The specific density for SPs was estimated at 1.11/100 000 and 0.66/100 000 for APs. However, this figure is higher than the previous estimate of 0.8/100 000 by Dahir and colleagues in 2020—although this estimate used the previous iteration of the LCoGS standards, including only *specialist* surgeons, anaesthetists or obstetricians rather than surgical, anaesthetic. or obstetric *providers*^3^.

## Discussion

A robust needs assessment is an important first step to improve any healthcare system, this is especially important in a low-income setting where resources are even more scarce^18^. This novel application of an existing tool to conduct a robust TNA has demonstrated that the Hennesy–Hicks training needs analysis (HHTNA) methodology is practical, both cost- and time-effective, and can develop potentially useful data even in a resource-constrained environment such as Somaliland. This is the first project to determine the educational needs of surgical and anaesthetic providers in Somaliland and the relevance of different procedures to their professional role. Identification of SPs and APs in Somaliland was facilitated by collaboration with a range of local organizations. There are multiple public, private, and charitable healthcare providers in Somaliland, many of which provide surgical services of varying complexity. The wide range of collaborating organizations provided access to providers across the country and allowed for relatively high participation in the project.

The survey has highlighted several important trends. Among APs, nearly 15% of respondents reported either no access or only infrequent access to intraoperative oxygen saturation monitoring. Additionally, only 51% of APs and 28% of SPs reported that they ‘always’ use the WHO Surgical Safety Checklist^14^. The HHTNA of APs highlighted that emergency FONA (cricothyroidotomy) was a ‘high intervention priority’ procedure among APs. Regional anaesthesia, medical management of co-morbidities, and anaesthesia in geriatric populations were also considered performance outliers and should also be the focus of further intervention. Mixed interventions were desired, indicating that training alone would be insufficient and that improvements to the work situation also need to be addressed.

Importantly, the numbers of SPs (69) and APs (41) identified in this study is significantly higher than the number of specialist surgeons (15) and anaesthetists (3) in previous work by Dahir *et al*.^3^. This highlights that surgical and anaesthetic task-sharing is commonplace in LMICs and reinforces the fact that any training or systems intervention targeted at the surgical workforce needs to be multidisciplinary by design or risk excluding the majority of clinicians who provide anaesthetic or surgical care^19^.

## Strengths and limitations

The overall number of responses in our study was modest, although in a country with a small healthcare workforce it was a reasonable response rate, especially from anaesthesia providers. It could be argued that the incomplete response rate introduced bias in the form of coverage error, sampling error, and non-response error^20^. The demographics of APs show that the responding cohort are relatively young, urban, and at the start of their careers, which likely adds some bias to the data reported, as the needs expressed may relate to the younger, urban members of the workforce and may not necessarily be generalized across the whole workforce. Therefore, there should be some caution applied when generalizing the results of this study to the wider population and particularly to the more senior and more rural members of the workforce. However, the response rates of 59% for APs, 33% for SPs, and 21% for obstetric providers are reasonable for survey-based research that often has a response rate of 35–45%^21,22^. This indicates that a significant proportion of the workforce have been included.

The Hennessy–Hicks methodology, although robust, does imply an arbitrary cut between when a skill is considered ‘satisfactory performance’ or ‘high intervention priority’—see *Figs 1*, *2*. This is set at the halfway point on the Likert scale of 1–5. Caution should be applied when interpreting this cut-off, and as such, skills that are approaching the halfway mark have been identified as ‘areas of concern’ in this study. Additionally, the performance data are self-reported and the accuracy could be improved through use of standardized metrics such as the Objective Surgical and Technical Skills; however, this would require significant extra resources^23^.

A further benefit of this project was through collaboration with colleagues in Somaliland. By jointly conducting a research project from initial proposal, including designing the survey and securing ethical approval through to data collection, analysis of the results and writing an academic article, local research capacity has also been strengthened.

## Conclusion

This study has demonstrated that conducting a pragmatic TNA of the surgical team in a low-resource setting, such as Somaliland, is feasible and can engage a significant proportion of the surgical workforce. It can also generate useful data that can potentially improve training and professional development. The development of a novel TNA tool, developed specifically for use among surgical teams, will facilitate performing a TNA of surgical teams in other locations. This is an essential pre-requisite for the future design of educational and training products, which are specifically relevant to the surgical workforce in LMICs.

## Supplementary Material

znaf216_Supplementary_Data

## Data Availability

All data collected are available on request by emailing the corresponding author.
