# Supplementary material for: Training needs analysis of surgical teams in Somaliland
Source: Br J Surg. 2025 Dec 10;112(Suppl 15):xv43–9. doi: 10.1093/bjs/znaf216 (PMC12690757; doi:10.1093/bjs/znaf216)
Supplement: znaf216_Supplementary_Data [file znaf216_supplementary_data.docx]

**Title:** A novel Training Needs Analysis of surgical teams in Somaliland.

**Authors:** Gerard McKnight^1,2,3,4^, Hassan Ali Daoud^5^, Rocco Friebel^1, 6^, Rachel Hargest^1,2,4^.

1 - Global Surgery Policy Unit,

LSE Health,

London School of Economics and Political Science,

London,

United Kingdom.

WC2A 2AE

2 - Humanitarian Surgery Initiative,

Global Affairs Department,

Royal College of Surgeons of England,

38-34 Lincoln’s Inn Fields,

London,

WC2A 3PE

United Kingdom.

3 – Academic Department of Military Surgery & Trauma,

Royal Centre for Defence Medicine,

Edgbaston,

Birmingham,

B15 2SQ

United Kingdom.

4 - School of Medicine,

Cardiff University,

Heath Park,

Cardiff,

CF14 4XN

UK.

5 – SUUBAN - Center for Health System Strengthening,

Jigjiga yar 00252,

Hargesia,

Somaliland.

6 – Department of Health Policy,

London School of Economics,

London,

WC2A 2AE,

United Kingdom

**Corresponding author.** Name and address **ORCID ID**; **Twitter**

Gerard McKnight

ORCID: 0000-0003-1546-7853

Twitter: @GerMcKnight

**Supplementary Materials - Index**

| **Supplementary Methods** |  |
| --- | --- |
| Good Reporting of A Mixed Methods Study (GRAMMS) guideline. | *Page 3* |
| **Supplementary Results** |  |
| **Figure 1.** Current use of technology and desire for future use amongst APs in Somaliland | *Page 4* |
| **Figure 2.** SPs response to the current use of technology and the desire for future use of technology (N=69). | *Page 5* |
| **Table 2**. Mean rating, standard deviation and significance of importance and performance of each skill with standard deviation, by APs. | *Page 6* |
| **Table 3** Mean rating, standard deviation and significance of importance of improving training and improving the work situation for each skill amongst APs. | *Page 7* |
| **Table 4.** Factors and their corresponding Cronbach's Alpha and standardised Alpha for each analysis. Note rating scale C asked 'How often do you perform this procedure per year?" so was excluded from the reliability analysis. | *Page 8* |
|  |  |

**Supplementary Methods**

| Item | Guideline | Paragraph reported |
| --- | --- | --- |
| 1 | Describe the justification for using a mixed methods approach to the research question | Methods |
| 2 | Describe the design in terms of the purpose, priority and sequence of methods | Methods |
| 3 | Describe each method in terms of sampling, data collection and analysis | Methods & Results |
| 4 | Describe where integration has occurred, how it has occurred and who has participated in it | Methods |
| 5 | Describe any limitation of one method associated with the present of the other method | Limitations |
| 6 | Describe any insights gained from mixing or integrating methods | Discussion & Conclusion |

**Supplementary Table 1**. Good Reporting of A Mixed Methods Study (GRAMMS) guideline.

**Supplementary Results**

**Supplementary Figure 1**. Current use of technology and desire for future use amongst APs in Somaliland (N=41).

**Supplementary Figure 2.** SPs response to the current use of technology and the desire for future use of technology (N=69).

.

**Supplementary Table 2.** Mean rating, standard deviation and significance of importance and performance of each skill with standard deviation, by Anaesthesia Providers (APs). N= 41

| **Skill** | **Mean importance (SD)** | **Mean performance (SD)** | **Difference** | **Significance (Two-sided)** |
| --- | --- | --- | --- | --- |
| General anaesthesia | 6.60 (0.83) | 5.66 (1.05) | 0.94 | <0.001 |
| Regional anaesthesia | 5.76 (1.64) | 4.34 (2.00) | 1.41 | <0.001 |
| Spinal anaesthesia | 6.50 (1.00) | 5.91 (1.11) | 0.59 | 0.056 |
| Endotracheal intubation | 6.45 (1.06) | 5.74 (1.21) | 0.72 | 0.08 |
| Emergency front of neck access (FONA or cricothyroidotomy) | 4.69 (2.31) | 2.68 (2.05) | 2.01 | <0.001 |
| Management of perioperative complications | 6.40 (1.11) | 5.26 (1.30) | 1.14 | <0.001 |
| Perioperative assessment of a patient undergoing surgery | 6.51 (1.09) | 6.06 (0.92) | 0.46 | 0.085 |
| Perioperative analgesia | 6.14 (1.18) | 5.46 (1.35) | 0.69 | 0.006 |
| Neonatal resuscitation | 6.41 (1.30) | 5.47 (1.35) | 0.94 | 0.002 |
| Paediatric anaesthesia | 6.18 (1.27) | 5.34 (1.23) | 0.83 | <0.001 |
| Geriatric anaesthesia | 5.62 (1.68) | 4.61 (1.63) | 1.00 | <0.001 |
| Medical management of co-morbidities | 5.50 (1.73) | 4.53 (1.62) | 0.97 | <0.001 |

**Supplementary Table 3** Mean rating, standard deviation and significance of importance of improving training and improving the work situation for each skill amongst Anaesthesia Providers (APs). N = 41

| **Skill** | **Mean training (SD)** | **Mean situation (SD)** | **Mean difference** | **Significance (Two-sided)** |
| --- | --- | --- | --- | --- |
| General anaesthesia | 5.50 (1.77) | 5.59 (1.61) | -0.09 | 0.993 |
| Regional anaesthesia | 5.35 (1.98) | 5.76 (1.60) | -0.41 | 0.57 |
| Spinal anaesthesia | 5.32 (1.45) | 5.64 (1.27) | -0.31 | 0.416 |
| Endotracheal intubation | 5.60 (1.81) | 5.71 (1.67) | -0.11 | 0.158 |
| FONA | 5.63 (2.14) | 5.38 (2.20) | 0.25 | 0.60 |
| Perioperative complications | 5.80 (1.27) | 5.50 (1.19) | 0.30 | 0.028 |
| Perioperative assessment | 5.51 (1.57) | 5.33 (1.71) | 0.18 | 0.077 |
| Perioperative analgesia | 5.34 (1.57) | 5.39 (1.57) | -0.05 | 0.697 |
| Neonatal resuscitation | 5.23 (1.88) | 5.28 (1.89) | -0.05 | 0.334 |
| Paediatric anaesthesia | 5.74 (1.24) | 5.81 (1.32) | -0.07 | 0.787 |
| Geriatric anaesthesia | 5.56 (1.38) | 5.68 (1.30) | -0.12 | 0.854 |
| Medical management of co-morbidities | 5.29 (1.16) | 5.61 (1.48) | -0.32 | 0.170 |

**Supplementary Table 4.** Factors and their corresponding Cronbach's Alpha and standardised Alpha for each analysis. Note rating scale C asked 'How often do you perform this procedure per year?" so was excluded from the reliability analysis.

| **Factor** | **Number of Items** | **Cronbach’s Alpha** | **Standardised Alpha** | **KMO** |
| --- | --- | --- | --- | --- |
| Scale A - Importance | | | | |
| 1 | 7 | 0.845 | 0.881 | 0.694 |
| 2 | 5 | 0.891 | 0.894 |  |
| Scale B - Performance | | | | |
| 1 | 7 | 0.88 | 0.898 | 0.698 |
| 2 | 3 | 0.832 | 0.829 |  |
| 3 | 2 | 0.532 | 0.532 |  |
| Scale D – Improving training | | | | |
| 1 | 6 | 0.878 | 0.880 | 0.745 |
| 2 | 6 | 0.674 | 0.730 |  |
| Scale E – Improving work situation | | | | |
| 1 | 7 | 0.866 | 0.865 | 0.645 |
| 2 | 3 | 0.706 | 0.755 |  |
